# Co-expression pan-network reveals genes involved in complex traits within maize pan-genome

**DOI:** 10.1186/s12870-022-03985-z

**Published:** 2022-12-19

**Authors:** H. Busra Cagirici, Carson M. Andorf, Taner Z. Sen

**Affiliations:** 1 US Department of Agriculture – Agricultural Research Service, Crop Improvement Genetics Research Unit, Western Regional Research Center, 800 Buchanan St, Albany, CA 94710 USA; 2 US Department of Agriculture – Agricultural Research Service, Corn Insects and Crop Genetics Research Unit, Iowa State University, Ames, IA 50011 USA; 3 Department of Computer Science, Iowa State University, Ames, IA 50011 USA; 4 Department of Bioengineering, University of California, Berkeley, CA 94720 USA

**Keywords:** Co-expression network, Pan-network, Maize, Pan-genome, GWAS, Complex traits, Tassel branch number, Starch

## Abstract

**Background:**

With the advances in the high throughput next generation sequencing technologies, genome-wide association studies (GWAS) have identified a large set of variants associated with complex phenotypic traits at a very fine scale. Despite the progress in GWAS, identification of genotype-phenotype relationship remains challenging in maize due to its nature with dozens of variants controlling the same trait. As the causal variations results in the change in expression, gene expression analyses carry a pivotal role in unraveling the transcriptional regulatory mechanisms behind the phenotypes.

**Results:**

To address these challenges, we incorporated the gene expression and GWAS-driven traits to extend the knowledge of genotype-phenotype relationships and transcriptional regulatory mechanisms behind the phenotypes. We constructed a large collection of gene co-expression networks and identified more than 2 million co-expressing gene pairs in the GWAS-driven pan-network which contains all the gene-pairs in individual genomes of the nested association mapping (NAM) population. We defined four sub-categories for the pan-network: (1) core-network contains the highest represented ~ 1% of the gene-pairs, (2) near-core network contains the next highest represented 1–5% of the gene-pairs, (3) private-network contains ~ 50% of the gene pairs that are unique to individual genomes, and (4) the dispensable-network contains the remaining 50–95% of the gene-pairs in the maize pan-genome. Strikingly, the private-network contained almost all the genes in the pan-network but lacked half of the interactions. We performed gene ontology (GO) enrichment analysis for the pan-, core-, and private- networks and compared the contributions of variants overlapping with genes and promoters to the GWAS-driven pan-network.

**Conclusions:**

Gene co-expression networks revealed meaningful information about groups of co-regulated genes that play a central role in regulatory processes. Pan-network approach enabled us to visualize the global view of the gene regulatory network for the studied system that could not be well inferred by the core-network alone.

**Supplementary Information:**

The online version contains supplementary material available at 10.1186/s12870-022-03985-z.

## Background

Maize is one of the most widely cultivated grains in the world, serving as a staple feed and food source [1]. The success of maize as a crop is due to a diverse genome that has gone through thousands of years of trait selection by indigenous people in Southern Mexico [2], followed recently by decades of hybridization and molecular breeding. In addition to being a major carbohydrate source, maize is a vital model organism for genetics studies [3, 4]. Maize has many advantages over other model organisms including the ease of creating controlled crosses and inbreds, extreme genetic diversity, easy to measure phenotypes, and a rich set of genetic and genomic resources [5, 6]. Diverse germplasms are available around the world, for example through the USDA-ARS, National Genetic Resources Program (https://www.ars-grin.gov/) and the International Maize and Wheat Improvement Center (CIMMYT - Mexico) [7].

With the advances in the high throughput next generation sequencing technologies, the current challenge in genomics studies has shifted from genotyping to the data processing and the mining for the valuable information from the fully assembled diverse populations. One such population is the maize nested association mapping (NAM). The NAM population contains 25 diverse founder inbred lines where each was crossed with the reference genome, B73 and selfed to generate 200 recombinant inbred lines per genotype [1, 8]. Using a common parent provides several agronomic benefits [9, 10] with the advantages of both linkage and association mapping for important phenotypic traits [11]. The NAM population studies provided a comprehensive set of polymorphisms for the founder inbred lines by genotyping and projecting these polymorphisms onto progeny based on low-density markers [11]. This allows genome-wide association studies (GWAS) to identify the variants associated with the phenotypic trait at a very fine scale. GWAS has been successfully applied to identify numerous agronomic and metabolic traits in maize, including drought tolerance [12, 13], salt tolerance [14], plant height [15–17], kernel weight [18], starch content [19], and many other traits of major importance.

Complex phenotypes are not only regulated by a single gene acting as a marker, but by a set of gene interactions that are often organized into various types of biological networks [20]. Maize genetics appears to favor an infinitesimal model where traits are regulated by a myriad of variants with small effects [21]. Often in other model plants, such as rice or Arabidopsis, only a few genes with large effects (rare allele model) exert control over the phenotypic traits that, in maize, are controlled by a cumulative effect of numerous variants. For example, flowering time in maize is influenced by over 30 small-effect variants [20], while in rice [22] and *Arabidopsis* [23] the trait is explained by a small number of large-effect variants. As a result, identification and annotation of causal variations remain challenging, despite the great progress in GWAS studies. Evidence suggests that causal variations result in the change in gene expression [24] and that the change in gene expression, in fact, significantly contributes to the phenotypic diversity [25]. Indeed, gene expression analyses carry a pivotal role in studying the function of genetic variation and are increasingly gaining a major role in unraveling the regulatory mechanisms of complex traits.

Network analysis based on gene expression similarities across different tissues and conditions aid the discovery of genes with regulatory importance [26]. Gene co-expression networks expose the co-regulated genes that share a similar expression pattern above a threshold across multiple conditions. Since genes under the same regulatory process tend to be related functionally [27], co-expression networks have been constructed to infer functional annotation of a gene and regulatory interactions between genes in *Arabidopsis*, rice, maize, and many other plant species [28–33]. On the other hand, co-expression network analysis has some caveats, for example, high false positive interactions mainly due to the absence of evidence for any physical or regulatory links, and limited set of interactions due to the expression and association cutoffs. Although not all functional interactions are captured, this network approach still provides highly informative evidence of gene interaction and can also provide valuable sources for the interpretation and validation of GWAS associated loci. In fact, a combination of gene expression network and GWAS analysis provides further confidence and reduce the false positive rate [34]. Recently, GWAS-integrated network analyses were used to enhance the biological interpretation and characterization of candidate causal genes to phenotypic variations in maize and other plants [33, 35–37].

A pan-genome approach was performed by Schaefer et al. to construct a ‘genotype’ network of maize seedlings across diverse maize genomes [33]. They showed that co-expression studies in general provide a powerful basis for candidate causal gene identification for GWAS loci, but the results are highly dependent on the gene expression data context. Another co-expression study evaluated the types of co-expression networks and showed that expression variation across pan-genomes in a single tissue/condition provide stronger evidence than the expression variation across different tissues in a single genome [36]. However, these studies did not look into the fact that co-expressing gene pairs across different conditions in a single genome might be conserved across different genomes.

In this study, we incorporated the phenotypic trait data into the co-expression networks such that the network represents only phenotypically important gene associations. To do so, we systematically compared 20 transcriptomic datasets for each of 26 maize NAM genomes individually to unravel the transcriptional regulatory mechanisms of the genes overlapping with a trait-associated loci. In contrast to the ‘genotype’ networks, we created co-expression networks for individual genomes based on the expression data across different tissues and applied a pan-genome approach to the 26 co-expression networks of diverse maize genomes. We applied pan- and core- network approaches to identify the atlas of transcriptional regulation and the basic regulatory mechanisms of traits, respectively. We provided GO enrichment analysis for the pan-, core-, and private- networks and compared the contributions of variants overlapping with genes and promoters to the GWAS-driven pan-network. Finally, as case studies, we demonstrated how the integration of GWAS data into a co-expression network allows us to better understand the mechanisms regulating the Tassel Branch Number and Starch traits.

## Results

### The construction of a large collection of GWAS-driven co-expression networks from maize NAM genotypes

We integrated GWAS data with the co-expression networks such that the network represents only phenotypically important gene relationships. GWAS-derived candidate genes associated with 41 diverse phenotypic traits [11] were selected to construct trait-specific co-expression networks. Based on the RNA-Seq expression values (TPM) across multiple samples for each genome, gene pairs with a significant co-expression relationship were determined based on Pearson Correlation (*r* > 0.9 and *p*-val < 0.001) and GWAS-driven co-expression networks were constructed for 26 maize NAM lines separately, including only the genes overlapping with a trait-associated SNP position within their genic or promoter regions. Each node denotes a gene in these networks and each edge connecting two nodes indicates a co-expression regulation. The number of genes (nodes) involved in these co-expression networks are similar for each genotype; however, the number of interactions (edges) varies considerably. On average, 5787 (+/− 4%) genes are involved in co-expression networks per genotype (Additional file 1: Table S1). The number of co-expressed pairs varies between 131,788 and 416,237 with an average of 230,620 co-expressed gene pairs per genotype, suggesting that trait-associated gene networks are drastically different from each other even though they contain similar number of genes.

### The construction of maize pan-network

**Fig. 1:**
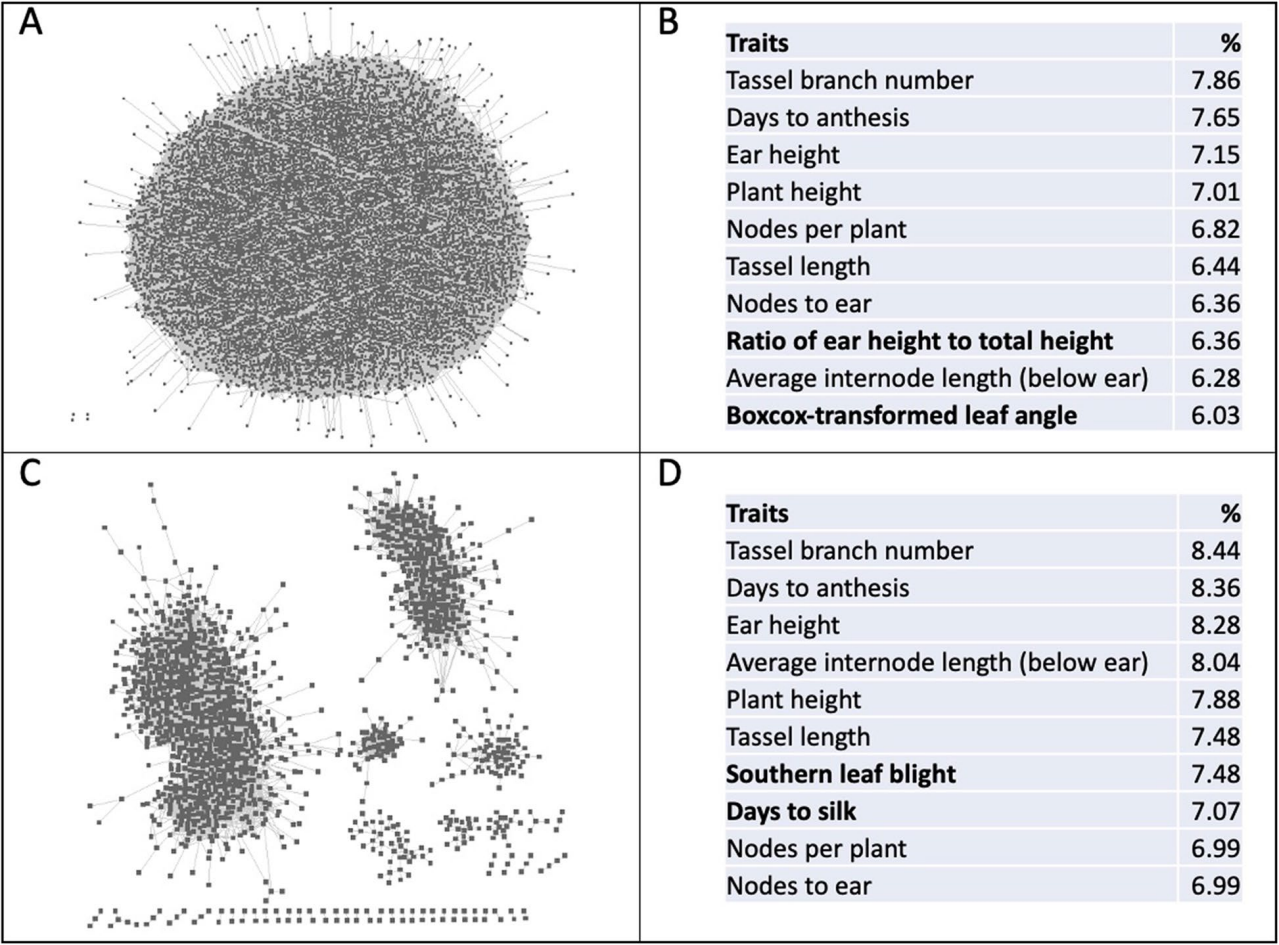
An overview of the GWAS-driven co-expression networks. **A** The collection of co-expressed gene-pairs in the pan-network and **B** the highest represented traits within the pan-network are shown at top. **C** The core-network and **D** the highest represented traits within the core network are shown at the bottom. Unique trait terms within the top 10 lists were bolded. Please see the text for the definitions of the networks

**Fig. 2:**
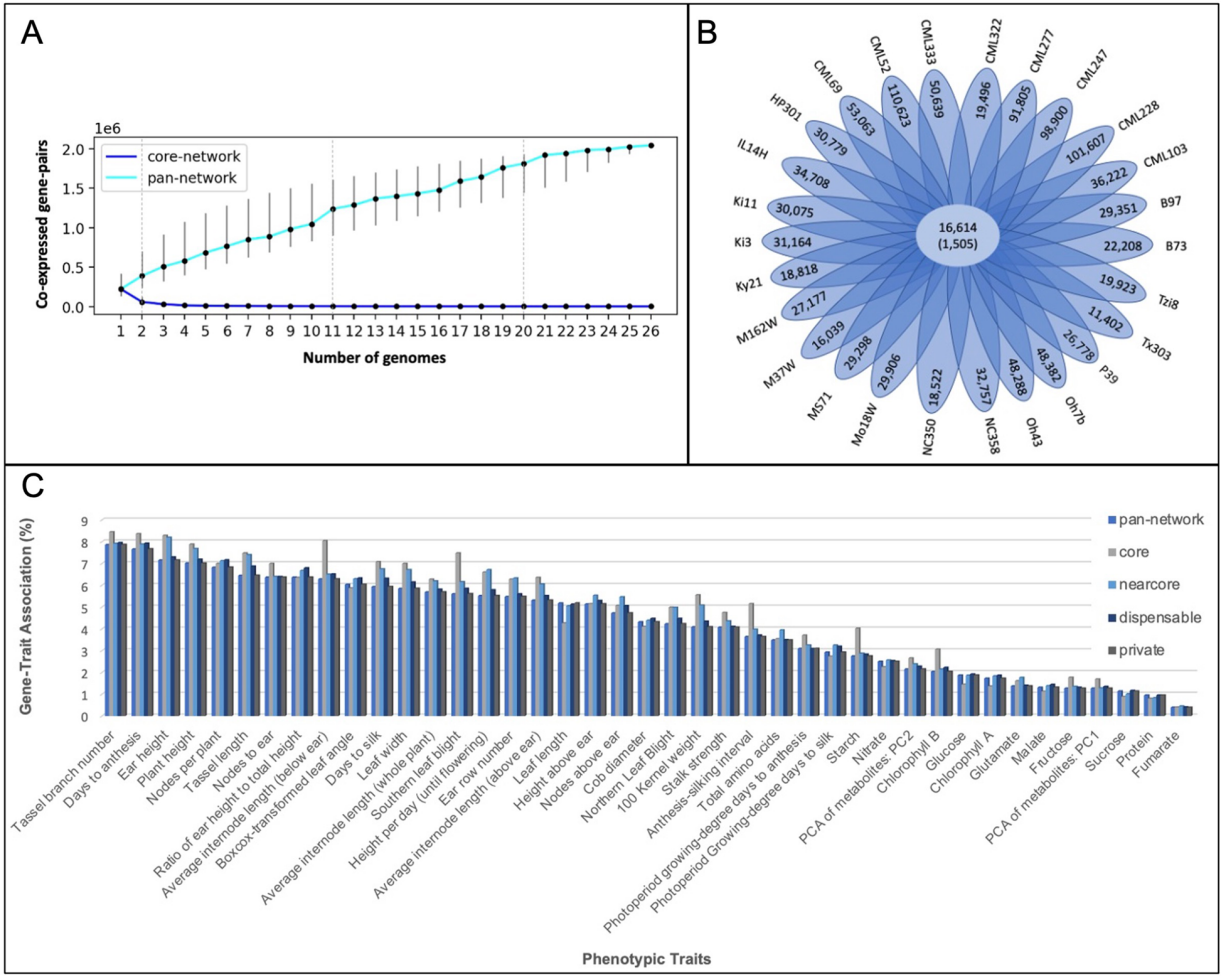
Pan-network composition revealing the distribution of co-expressed gene-pairs among the NAM genomes and the gene-trait associations. **A** The growth curve of the pan- and core- networks based on the number of co-expressed gene-pairs. The continuous cyan curve represents the total number of co-expressed gene-pairs for a given number of genomes, the blue curve indicates the number of core gene-pairs, and the vertical bars indicate the standard deviations. Dashed lines, separating the plot into four sections, indicate the sub-categories of pan-network, as core-, dispensable-, near-core-, and core- networks, respectively. **B** Venn diagram illustrating the number of co-expressed gene-pairs common and unique to the genomes specified. The size of the core-network is shown in the center as the number of co-expressed gene-pairs and the number of genes inside the brackets. The leaves represent the number of unique gene-pairs found in each genome. **C** Gene-trait association across pan-, core-, near core-, dispensable-, and private- networks. The proportion of the genes associated with the traits across the pan- and core- networks is shown per trait (please see the text for the definition of these categories)

A maize pan-genome co-expression network was constructed for the genes associated with a complex trait as defined in the Wallace GWAS data [11]. From the union of 26 co-expression networks, a ‘pan-network’ was constructed for the maize pan-genome. A global view of the network is shown in Fig. 1. The pan-network is an atlas of highly connected genes whereas the core-network is composed of specialized modules showing the core/main gene interactions. As shown in Additional file 2: Data S1, the GWAS-driven maize pan-network consists of 2,041,983 co-expression pairs among which 52% belong to private genotypes and only 0.04% are present in all 26 NAM genotypes (Fig. 2). A similar observation was reported for Arabidopsis where no co-expression pairs were detected in all the genotypes [26].

### Defining pan-network sub-categories

We classified the co-expression pairs in a pan-network into four sub-categories: private, dispensable, near-core, and core (Fig. 2A). The private network is defined by the co-expression pairs that are unique to one genome while 50 to 95% of the co-expression pairs observed in two to 10 genomes were grouped into the dispensable network (Additional file 2: Data S1). Instead of a strict definition of “core” where the genes are shared by every genotype [1], we adopted an extended core definition such that the highest represented co-expression pairs (the top 1%) were classified into the core-network and the ones present in the top 1 to 5% of the genomes were classified into the near-core network (Fig. 2A). Even with the extended definition, 99% of the co-expressed gene-pairs and 84% of the pan-network genes were not retained in the core network.

### Private network contains the same number of genes but lacks some of the interactions

Co-expressed gene-pairs and gene interactions vary greatly among the NAM genomes. Our results show that half of the interactions in the pan-network were unique to a specific genome. We observed a great variance in the private gene-pairs across the genomes. For example, more than 25% of the co-expressed pairs in CML228, CML247, CML277, and CML52 genotypes were private while private interactions were less than 10% in CML322 and Tx303 genotypes (Fig. 2B and Additional file 2: Data S1). We did not observe any difference in the experimental settings and geographic origin to differentiate the variations in the proportion of the unique gene-pairs observed in these genomes. Strikingly, although the private network was missing half of the gene-pair interactions in the pan-network, it included almost all the genes (Table 1). This might be attributable to the fact that the traits are regulated by the accumulative effect of the genes rather than a few regulatory genes themselves. These genes might be involved in different transcriptional regulatory mechanisms rather than interacting with the same set of genes.

**Table 1:** The number of genes and co-expressed gene pairs across trait-associated pan-network categories. Co-expression pairs in a pan-network into four sub-categories: private, dispensable, near-core, and core. The private network is defined by the co-expression pairs that are unique to one genome while 50 to 95% of the co-expression pairs observed in two to 10 genomes were grouped into the dispensable network. The highest represented co-expression pairs (the top 1%) were classified into the core-network and the ones present in the top 1 to 5% of the genomes were classified into the near-core network (please see text for more detailed explanation)

	Pan-network	Pan-network sub-categories			
	whole	core	near-core	dispensable	private
**genes**	10,163	1505	3328	8154	10,162
**gene-pairs**	2,041,983	16,614	89,362	868,077	1,067,930

### Enriched biological functions in pan-network categories

The classification of pan- and core- networks can help identify all possible gene associations involved in the transcriptional process and infer the common gene associations required for a general regulatory function, respectively. Pan-network represents the extensive set of genes and co-expressed gene-pairs that might be involved in transcriptional regulatory processes. The core-network, on the other hand, represents the group of consistent genes and gene-pairs among multiple genotypes, thus, could employ the primary regulatory machinery to manipulate normal cellular function.

We investigated the biological significance of the pan-network categories through gene ontology (GO) enrichment analysis. We examined the diversity of the pan-, core-, near-core-, dispensable-, and private- networks, regarding the predominant biological processes involved in the trait-associated co-expression networks. The whole pan-network was enriched with 121 GO terms for the 3957 genes with GO annotations (FDR < 0.05). The pan-network categories showed various enrichment levels with 77, 104, 70, 121 GO terms in the core-, near-core-, dispensable-, and private- networks, respectively. A total of 219 enriched GO terms were identified from the different pan-network categories. In total, 59 GO terms overlapped the core- and near-core- networks while 69 of the 70 GO terms in dispensable network were in common with the private network. Core/near-core networks share less than 25% of the GO terms with the private/dispensable networks. As expected from the core/near-core networks, the enriched GO terms were mostly relevant to the key biological processes including biosynthetic processes, ATPase activity, chromatin modification, and histone acetylation (Additional file 3: Data S2).

### Genic regions regulate half of the trait associated gene co-expression networks

We examined the contribution of the variants within the genic regions alone to the co-expression networks. A separate pan-network was constructed for the co-expressed gene pairs overlapping with a trait-associated SNP within the genic regions. 12,090 SNPs were identified within the genic regions of 8894 genes. Pearson correlation analysis revealed that 5287 of these genes involved in the genic pan-network resulting in 663,494 gene-associations as opposed to 10,163 genes and 2,041,983 gene-associations in the genic/promoter pan-network (Additional file 4: Table S2). Our results showed that over half (52%) of the genes were originated from the genic pan-network whereas only 32% of the gene-associations were covered in the genic pan-network. The proportion of variance was similar among the pan-network sub-categories with 52 to 54% of the genes and 31 to 34% of the gene-associations were derived from the genic pan-network (Additional file 4: Table S2). Similar to the gene/promoter pan-network categories, 1 and 51% of the gene associations were involved in the genic core- and private- networks, respectively.

In terms of representation of the traits among the pan-network, gene/promoter and genic pan-networks shared a similar trait content. The highest represented traits within pan-networks were similar between gene/promoter and genic alone, only the traits Days to Silk and Plant Height showed a greater abundance over Tassel Branch Number and Ear Height in the genic pan-network. Overall, 44% of the traits were stemmed from genic pan-networks whereas 36% of the Protein and Chlorophyll B traits and 49% of the Height per Day (until flowering) trait were represented in the genic pan-network (Additional file 5: Data S3).

We performed GO term enrichment for the genic pan-networks and compared the enriched GO terms with the gene/promoter pan-network. A total of 79 GO terms were enriched in the genic pan-networks where 63 of the enriched GO terms were common between the gene/promoter and genic pan-networks and 16 GO terms were only enriched in the genic pan-networks (Additional file 6: Data S4). Genic network specific terms include major biological processes and molecular functions including regulation of cell division, regulation of cytokinesis, and regulation of cytokinetic process.

### Integration of GWAS loci to co-expression networks could specify candidate genes

Genome-wide association studies (GWAS) benefit from the high diversity in maize and provide the opportunity to study the genetic structure of the complex phenotypic traits at a very fine scale. Here, we integrated GWAS data for 41 agronomic traits, including both developmental and metabolic traits, into the co-expression networks to identify the regulatory interactions altering plant phenotype and investigated the contribution of pan-, core- and private- networks to the biological regulation of the traits (Additional file 9). The highest represented traits in the pan- and core- networks are shown in Fig. 1. Overall, 8 of the top 10 represented traits were common within the pan- and core- networks, including Tassel Branch Number and Days to Anthesis. Subsequently, the proportion of the genes associated with a trait in pan-network categories was extracted for each trait (Fig. 2C). The four traits, Tassel Branch Number, Days to Anthesis, Ear Height, and Plant Height were among the highest represented traits by the gene co-expression pairs in both pan- and core- networks. The abundance of each trait in pan- and core- networks were relatively similar, except a few. Starch and Chlorophyll B traits showed a larger abundance in the core-network as well as the Average Internode Length (below ear), Southern Leaf Blight, and Anthesis-silking Interval traits. As a case study, we selected two traits: specifically, one developmental trait and one metabolic trait. However, the list of co-expression pairs for individual traits is provided in Additional file 7: Data S5 together with information regarding the individual genomes representing the co-expression pair, total number of genomes the co-expression pair are observed, and the pan-genome sub-category of the co-expression pair. The first trait, the Tassel Branch Number, is the most represented trait in both the pan- and core- networks. The second trait, Starch, is one of the metabolic traits. Although the number of SNPs associated with Starch, and thus the number of genes and gene-pairs, are smaller, the gene pairs associated with the Starch trait were more abundant in the core-network.

### Tassel branch number

**Fig. 3:**
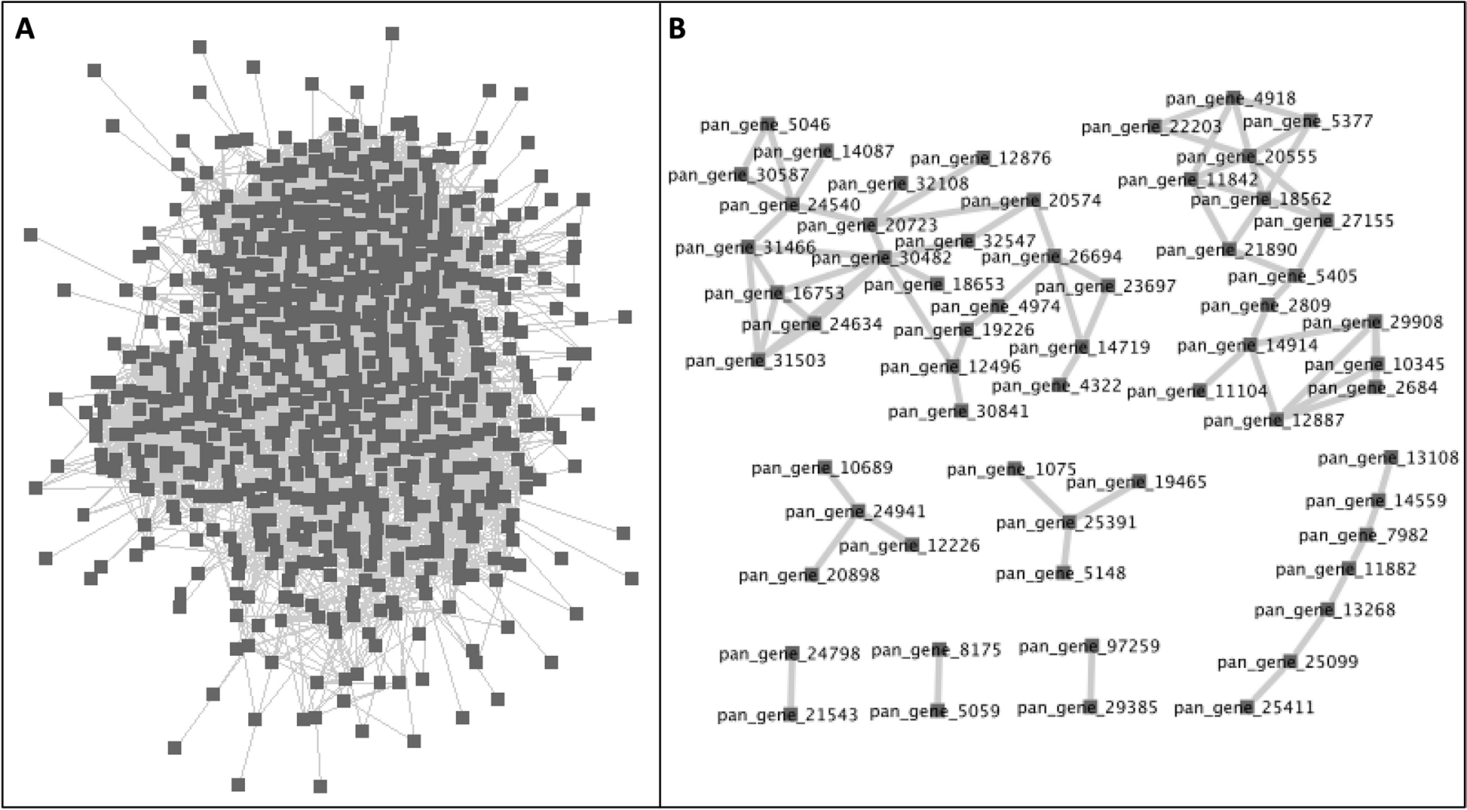
The co-expression networks associated with the Tassel Branch Number (TBN) as (**A**) the pan-network and (**B**) the core-network. Each node represents a gene associated with TBN and each edge represents a co-expressed gene pair. The pan-network contained 697 genes and 10,718 co-expression pairs, assembling into one large regulatory network. In contrast, core-network was substantially smaller with 60 genes and 74 gene-pairs and was composed of 8 sub-networks

We investigated the transcriptional regulation of the Tassel Branch Number (TBN) trait using the pan- and core- networks. The TBN is an important agronomic trait contributing to yield and it has been increasingly investigated in maize breeding programs. Most complex traits such as the TBN are controlled by numerous minor-effect variant in maize [10, 20, 38]. Since the majority of the variants have small effects on the phenotype, they cannot be easily incorporated into maize breeding programs. Thus, identification of TBN controlling variants and their regulatory mechanisms would provide useful information to facilitate high-yield maize breeding. Here, we aim to identify the transcriptional regulatory mechanisms controlling TBN and, by leveraging a pan- and core- network approach, we aim to gain valuable insights into the global and core components regulating the TBN (Fig. 3). The pan-network contained 697 genes and 10,718 co-expression pairs, assembling into one large regulatory network (Fig. 3A). In contrast, core-network was substantially smaller with 60 genes and 74 gene-pairs and was composed of 8 sub-networks (Fig. 3B).

The core-network is considered a reliable network because the interactions are supported by a large number of genomes, making it a primary network that represents the basic regulatory mechanisms associated with the trait. Accordingly, we constructed a trait-specific core-network for the TBN-associated genes, reflecting the co-expression of TBN-associated genes to genes near other loci associated with the same trait. These genes were defined by the degrees of associations and the genes with the highest degrees of associations were denoted as hub genes. Thus, the degree of association reflects the impact of a gene on the overall network. The highest degree of association was observed for a Cyclin-dependent kinase (pan_gene_30482) with eight neighbor genes. This hub gene was co-expressed with an ARID-transcription factor gene (pan_gene_20723) with five neighboring genes. Co-expression edges between kinases and transcription factors are of particular biological interest as they may indicate gene regulation triggered by signaling pathways. Another hub gene with six associations was an oxygen evolving complex (pan_gene_20555). The core-network, overall, contained eight transcription factors and four kinases. Seven of the 60 genes in the core-network were not annotated either with InterPro or GO terms. GO terms assigned to the core-network included variety of biological processes and molecular functions, such as DNA-directed 5′-3′ RNA polymerase activity, response to oxidative stress, protein binding, and GTPase activity. However, no GO terms were significantly enriched in the core-network.

In contrast with core-network, pan-network illustrated the atlas of transcriptional regulation of TBN associated genes (Fig. 3B). According to pan-network of TBN associated genes, Cyclin-dependent kinase (pan_gene_30482) was also highly regulated by genes in TBN pathway with a large set of correlated genes (132 neighbor genes). Similarly, ARID-transcription factor gene (pan_gene_20723) presented one of the highest degrees of association (131 neighbor genes) for the TBN regulatory mechanism in the pan-network. Contrary to the core-network, pan-network showed enrichment for six GO terms: binding, protein binding, two-component sensor activity, phosphotransferase activity, protein histidine kinase activity, and riboflavin synthase complex. Overall, the pan-network provided a picture of global gene regulatory mechanism for the TBN that could not be well inferred by the core-network.

### Starch metabolism

Maize provides a rich source of calories since ~ 70% of the weight is carbohydrates, mostly in the form of starch [39]. Starch is among the complex traits controlled by many genes, thus offering multiple gene targets to facilitate crop yields and end-use quality [40, 41]. Therefore, understanding the mechanism of starch metabolism and regulation is critically important for meeting future needs. The Wallace GWAS dataset contains trait data for 12 metabolites including starch from the leaves of maize. Although Starch was not among the top represented traits in either pan- or core- networks, the percentage of genes associated with the Starch over the 41 traits represented in the core-network is larger than in the pan-network (Fig. 2C).

We constructed pan- and core- networks for the Starch trait. The Starch pan-network contains 245 genes and 1433 gene-pairs while the core-network is composed of 18 genes and 17 gene-pairs (Table 2 and Additional file 7: Data S5). Of the 18 genes in the core-network, 12 were assigned with at least one GO term, which includes ATP hydrolysis activity, GTPase activity, oxidoreductase activity, and 1;3-beta-D-glucan synthase activity. Among the 245 genes in pan-network, 34 were unannotated in the InterPro database and 88 were unannotated in the GO database. Annotated genes were assigned with a wide range of GO terms, including four genes assigned with carbohydrate metabolic process (GO:0005975) and four genes with glycosyltransferase activity (GO:0016757). AMP-activated protein kinase, Alpha-amylase/branching enzyme, and Sucrose synthase were among the functional annotation of the genes involved in the Starch pan-network. A detailed list of GO and InterPro annotations for Starch pan- and core- networks is available in Additional file 8: Data S6.

**Table 2:** Trait-specific co-expression network statistics for pan-network categories including pan-network, core-, near core- dispensable-, private- networks. Subcategory definitions can be found in the text and in the caption of Table 1. The pan category contains all pairs. “N” stands for nodes of a network, in this case genes, and “e” stands for edges of a network, in this case gene-pairs

	Core		Near-core		Dispensable		Private		Pan	
Traits	n	e	n	e	n	e	n	e	n	e
100 Kernel weight	22	16	70	134	255	1343	361	1619	363	3112
Anthesis silking interval	18	22	72	149	234	1035	337	1258	339	2464
Average internode length (above ear)	22	25	95	236	306	2078	451	2754	451	5093
Average internode length (below ear)	48	64	122	331	397	2938	557	3634	561	6967
Average internode length (whole plant)	36	50	108	226	389	2464	542	3255	543	5995
Boxcox-transformed leaf angle	46	51	115	240	410	2555	566	3616	568	6462
Chlorophyll A	5	3	15	12	96	215	147	316	151	546
Chlorophyll B	22	21	36	58	111	355	169	453	171	887
Cob diameter	33	41	86	193	270	1667	340	1960	343	3861
Days to anthesis	55	97	145	332	469	4510	642	5218	644	10,157
Days to silk	34	29	107	203	350	2812	491	3256	491	6300
Ear height	54	71	144	374	501	4231	660	5142	661	9818
Ear row number	39	36	115	202	344	2189	482	2662	482	5089
Fructose	0	0	11	8	61	99	85	114	95	221
Fumarate	0	0	2	1	16	13	18	15	24	29
Glucose	0	0	22	14	102	256	139	338	146	608
Glutamate	0	0	15	13	74	174	100	187	108	374
Height above ear	35	28	94	170	312	1757	414	2087	418	4042
Height per day (until flowering)	33	29	118	222	351	2412	485	2861	486	5524
Leaf length	43	44	119	273	363	2049	493	2767	494	5133
Leaf width	45	79	112	246	380	2626	518	3161	522	6112
Malate	2	1	15	11	61	137	106	163	111	312
Nitrate	16	17	37	51	118	512	175	514	179	1094
Nodes above ear	19	15	86	143	297	1528	451	2297	452	3983
Nodes per plant	47	58	117	316	448	3722	604	4457	605	8553
Nodes to ear	41	45	114	241	386	3201	517	3753	519	7240
Northern Leaf Blight	23	19	81	134	260	1503	368	2046	369	3702
PCA of metabolites: PC1	4	2	13	10	58	133	92	174	96	319
PCA of metabolites: PC2	8	6	33	31	138	453	204	617	207	1107
Photoperiod growing-degree days to anthesis	21	26	54	113	174	714	248	763	253	1582
Photoperiod growing-degree days to silk	23	13	57	92	158	850	217	791	224	1780
Plant height	45	46	144	346	451	4157	615	5007	616	9556
Protein	0	0	10	5	32	47	65	71	70	123
Ratio of ear height to total height	47	61	124	342	403	3081	564	3642	564	7126
Southern leaf blight	40	42	118	279	343	2388	461	3002	461	5711
Stalk strength	33	33	76	151	222	1239	305	1338	309	2761
Starch	18	17	44	44	170	599	241	773	245	1433
Sucrose	2	1	11	7	53	78	76	129	83	215
Tassel branch number	60	74	145	436	501	4353	696	5855	697	10,718
Tassel length	44	42	130	283	424	3082	578	4075	578	7482
Total amino acids	10	6	53	79	190	763	289	1088	292	1936

*n: nodes and e: edges

One gene can contribute to more than one phenotypic trait and is referred to as a pleiotropic gene. Pleiotropic effects have been reported in maize for various traits. Here, we investigated the pleiotropic effects of the genes associated with Starch. We observed that 161 of the genes in Starch pan-network are pleiotropic genes associated with two to eight nonredundant phenotypic traits. We also observed the associations with other agronomic traits (Table 3), such as Plant Height and Ear Height, suggesting that the starch pathway is linked to other traits important for yield. In fact, the yield is largely determined by the starch content in maize [40, 41]. Possibly, improving the starch content could lead to higher-yield products by targeting the genes regulating more than one trait.

**Table 3:** The number of pleiotropic genes associated with Starch that are also associated with other phenotypic traits. 161 of the genes in Starch pan-network are pleiotropic genes associated with two to eight nonredundant phenotypic traits. Only top 10 traits were shown in the Table

Top 10 traits linked with the Starch trait	Gene count
PCA of metabolites: PC2	23
Nodes per plant	19
Nodes to ear	19
Plant height	16
Boxcox-transformed leaf angle	15
Ear row number	13
Nodes above ear	11
Tassel length	11
Average internode length (wp^a^)	10
Ear height	10

^a^whole plant

## Discussion

The advent of whole genome sequencing technologies and genome-wide profiling experiments stimulated the progress in identifying loci associated with complex traits, enabling linking genotypic variations with phenotypic changes. However, revealing phenotype-genotype associations solely are insufficient since such associations do not uncover the molecular mechanisms underlying it. Especially for species like maize, where multiple rare variants often regulate complex traits, discovering the co-regulation of trait-associated loci is of critical importance to understand the molecular pathways regulating the trait. Additionally, there is the issue of the heritability of such associations. Therefore, advanced methods are required to investigate phenotype-genotype associations in terms of the molecular pathways involved. Gene co-expression networks are often opted to reveal important associations between genes and represent the co-regulated genes that play a central role in regulatory processes. As such, we incorporated the GWAS data into the co-expression networks to unravel the transcriptional regulatory mechanisms behind these phenotypic traits at the pan-genome level.

Genes exhibiting coordinated expression across samples are likely to be biologically co-regulated. Thus, co-expression networks have the potential to infer the regulatory network of genes and, together with GWAS data, co-expression studies could reveal the effects of regulatory networks to important phenotypic traits that are of high agronomic and biological importance. We constructed co-expression networks for the genes overlapping with a trait associated loci based on expression similarities across samples for each maize NAM genome. Similar to a pan-genome approach, we investigated the co-expressed gene-pairs at the pan- and core- network levels. The pan-network represented the entire set of co-expressed gene-pairs for the 26 maize NAM genotypes. The pan-network was composed of the core-network, which corresponds to the highest represented 1% of the co-expressed pairs, the near-core-network, which covers the highest 1 to 5% of the co-expressed gene pairs, the private-network, which includes the gene-pairs co-expressed in only one genotype, and the dispensable-network, where the co-expressed gene-pairs are absent in several genotypes. This study showed strikingly that although most of the genes are in fact core genes, only ~ 1% of the co-expressed gene-pairs were in the core-network. This finding suggests that in a pan-genome approach, classification of pan- and core- are different for genes and gene pair interactions and although genes may be conserved across NAM genomes, their transcriptional regulation may not. A similar observation was reported for Arabidopsis pan-network where no co-expression pairs were detected in all the genotypes [26].

Similar co-expression studies using the same inbred genomes were performed in maize before [33, 36]; however, our definition of pan-network differs from the earlier maize co-expression studies. The Co-expression Browser (COB) and Camoco approaches integrated different GWAS data to the maize co-expression networks using the same inbred genomes; however, these co-expression networks were ‘genotype’ networks based on a one tissue/condition expression profiles across diverse maize genomes [33, 36]. Instead, we created single-accession co-expression networks for each genome and created the pan-network from the union of 26 individual co-expression networks for each genome. The benefit of our approach is we could differentiate private interactions specific to a genome and the core interactions conserved in most genomes.

To date, GWAS has been successfully applied to identify variants associated with numerous traits in maize including ionomic, developmental, adaptive/stress response, and metabolic traits [36, 42]. The co-expression analysis has been applied to unravel the biological mechanisms driving the associated traits and the candidate causal genes leading to the phenotype for many plants including Arabidopsis and maize. Integration of GWAS data into a co-expression network provided evidence for identifying important genes associated with the oil-related traits [42]. A larger scale analysis was performed later to determine the high-priority candidate causal genes under ionomic GWAS loci [36].

The focus of this study was to unravel the transcriptional regulatory mechanisms for the 41 agronomic and developmental phenotypic traits. We provided a large set of co-expressed gene-pairs as well as co-expression networks for trait-associated loci so that the data could be of use for further studies. We provided functional annotation of genes involved in these networks, GO categories, and the enriched GO terms.

We provided examples of the use of co-expression networks for the transcriptional regulation of complex traits, such as Tassel Branch Number and Starch. Both Tassel Branch Number and leaf starch content are among the complex agronomic and metabolic traits, respectively, and are controlled by many genes. Since majority of the variants have small effects on the phenotype, they cannot be easily incorporated into the maize breeding programs. Thus, understanding the underlying mechanism of these traits and regulation is critically important to facilitate high-quality and high-yield maize breeding for meeting future needs. Overall, the pan-network approach provides an enhanced global picture of the gene regulatory network for a studied system that could not be well inferred by core-network of genes alone.

## Conclusions

In this study, we provided a large collection of co-expressing genes in the GWAS-driven pan-network for maize NAM genomes. By incorporating the phenotypic trait data into the co-expression networks, we aimed to reveal phenotypically important gene associations. We demonstrated how the integration of GWAS data into a co-expression network allows us to better understand the mechanisms regulating the complex traits in maize. We provided pan-networks specific to 41 agronomically important traits so that the data could be of use for further studies.

## Methods

### The pan-genome and annotations

The latest B73 RefGen_v5 reference genome and the 25 NAM founder genomes (collectively referred to as the 26 NAM lines) and annotations were retrieved from Hufford et al. (2021) through MaizeGDB [3]. We included the pan-gene annotations rather than canonical annotations to cover the pan-genes that exist in a genome but were not annotated. A total of 103,538 pan-genes was included for 26 NAM genomes. The mappings of InterPro entries to Gene Ontology (GO) terms were retrieved from the InterPro protein families and domain database [43]. GO terms for the pan-genes were extracted from the InterPro annotations provided within the GFF3 annotation files of NAM genomes.

### The GWAS data

We collected the list of single nucleotide polymorphisms (SNPs) that were associated with 41 phenotypic traits reported previously from a GWAS study by Wallace et al. (2014). In general, the data for 41 traits was linked with the high-confidence SNP markers across NAM by fitting a joint-linkage model. A matrix of 35,770 GWAS SNP positions in each genome and associated traits is available in the CyVerse database [44] and provided as Data S7. To extract the pan-genes associated with a phenotypic trait, GWAS-hits were intersected with the gene annotations for individual genomes using bedtools intersect function. We included the pan-genes overlapping with a trait associated loci within their annotated genic regions and promoters (< 5 kb upstream) (Table S1). A pan-gene can overlap with multiple GWAS-hits. Nonredundant trait annotations were assigned to pan-genes for each GWAS-hit.

### RNA-Seq data

All the expression datasets in this study were retrieved from the Maize NAM Consortium [1]. The Maize NAM Consortium sequenced 20 samples for each NAM genome and expression data is represented as transcripts per million mapped reads (TPM). The TPM values from RNA-Seq data for the NAM genomes are available through the CyVerse Data Commons database. The TPM data is available for ten tissues, including: (1) primary root, (2) shoot at 6 days after planting, (3) base of the 10th leaf, (4) middle of the 10th leaf, (5) tip of the 10th leaf, (6) meiotic tassel, (7) immature ear at the V18 growth stage, (8) anthers at the Reproductive 1(R1) growth stage, (9) endosperm, and (10) embryo at 16 days after pollination. With a few exceptions, two biological replicates for each tissue resulted in 20 RNA-Seq samples for individual NAM genomes and thus resulted in 103,538 genes × 20 RNA-Seq samples gene-expression matrix for each of the 26 genomes. For each genome, genes with median TPM lower than 5 were excluded for noise attenuation to create the high-confidence list for downstream. After filtering, 33,021 of the 103,538 pan-genes that have at least 5 median TPM in a genome were included in our study.

### Construction of co-expression networks

The collection of gene expression data from the tissue samples for each NAM genome were used for the construction of co-expression networks. An unsigned co-expression network was inferred by a pairwise Pearson correlation using scipy.stats module in the Python SciPy library. A strict significance threshold (R cut-off of 0.9 and significance threshold of *P* = 0.01) was applied to select the significant co-expressed gene-pairs. Individual co-expression networks were constructed for 26 NAM genomes. The union of co-expression pairs in 26 NAM genomes was defined as the ‘pan-network’ and the co-expression pairs specific to an individual genome was defined as ‘private network’. All interaction networks were visualized using Cytoscape (v3.8.0) [45].

### Enrichment analysis

To explore the biological significance of the co-expression networks, we performed functional enrichment analysis for GO annotations using the BiNGO plugin [46] for Cytoscape (v3.8.0). The default statistical parameters were applied for the hypergeometrical statistical test along with a Benjamini and Hochberg false discovery rate (FDR) correction at a significance level of 0.05. The enrichment analyses in the pan-network (high-confidence data) were performed using high-coverage data as background unless specified otherwise.

## Supplementary Information

**Additional file 1 Table S1.** GWAS-hits within genes and promoters (TPM > 5 median).

**Additional file 2 Dataset S1.** The number of edges shared by the number of lines. The column “number of lines” represent the number of co-expressed gene-pairs (edges) observed in the number of lines. For example, the 5th row represents the unique gene-pairs for each genome whereas the row 30th represent the gene-pairs observed in all 26 NAM genomes.

**Additional file 3 Dataset S2.** Enriched GO terms in pan-network sub-categories.

**Additional file 4 Table S2.** genic vs genic/promoter network stats.

**Additional file 5 Dataset S3.** The distribution of 41 complex traits within the genic and genic/promoter networks.

**Additional file 6 Dataset S4.** GO term enrichment in the genic and genic/promoter networks.

**Additional file 7.**

**Additional file 8 Dataset S6.** Starch associated genes and annotations.

**Additional file 9.**

## Data Availability

All data generated during this study are included in this published article and its supplementary information files. The datasets generated and/or analyzed during the current study are available in the figshare repository at doi.org/10.6084/m9.figshare.21357912.

## Abbreviations

GWAS: genome-wide association studies
NAM: nested association mapping
GO: gene ontology
CIMMYT: International Maize and Wheat Improvement Center
TBN: Tassel Branch Number
TPM: transcripts per million mapped reads
COB: Co-expression Browser
SNPs: single nucleotide polymorphisms
FDR: false discovery rate
